# A Boolean network control algorithm guided by forward dynamic programming

**DOI:** 10.1371/journal.pone.0215449

**Published:** 2019-05-02

**Authors:** Mohammad Moradi, Sama Goliaei, Mohammad-Hadi Foroughmand-Araabi

**Affiliations:** 1 Faculty of New Sciences & Technologies, University of Tehran, Tehran, Iran; 2 Department of Mathematical Sciences, Sharif University of Technology, Tehran, Iran; University of Aveiro, NEW ZEALAND

## Abstract

Control problem in a biological system is the problem of finding an interventional policy for changing the state of the biological system from an undesirable state, e.g. disease, into a desirable healthy state. Boolean networks are utilized as a mathematical model for gene regulatory networks. This paper provides an algorithm to solve the control problem in Boolean networks. The proposed algorithm is implemented and applied on two biological systems: T-cell receptor network and Drosophila melanogaster network. Results show that the proposed algorithm works faster in solving the control problem over these networks, while having similar accuracy, in comparison to previous exact methods. Source code and a simple web service of the proposed algorithm is available at http://goliaei.ir/net-control/www/.

## 1 Introduction

A gene regulatory network (GRN) is a mathematical model of genes and their interaction [1]. The purpose of GRN studies is to achieve a new insight toward the important cellular processes. Examples of mathematical modeling of biological processes includes cell cycle [2, 3], oscillations in p53-mdm2 system [4–6], phage-lambda system [7–9], and T-cell large granular lymphocyte (T-LGL) leukemia network [10, 11]. There are different techniques for modeling dynamics of GRNs, including Boolean networks (BNs) [11], Bayesian networks [11], dynamic Bayesian networks [11], linear models [12] and differential equations [12]. Among the above mentioned models, the Boolean network model has received many attentions [13–16]; that is because in addition to the tractability, Boolean networks could be reconstructed by efficient biological experiments [17, 18].

Detecting a set of perturbations which cause the desirable changes in cellular behavior has many applications such as cancer treatment and drug discovery [5, 19–23]. This highlights the necessity of developing a control theory for the gene regulatory networks. Using GRN control model is considered as a key method to design the experimental control policies [23].

The control problem includes finding a sequence of interventions to be applied on the system, which changes state of the system from an undesirable state of the network to a desirable one [24–26]. The undesirable state in a gene regulatory network may express a disease such as cancer, and the desirable state can express the wellness, for example as induction of apoptosis in cancerous cells or tumors. Therefore, using the case of control and medical interventions, we can exterminate the tumor cells and achieve healthiness [26].

### 1.1 Related works

Up to now, numerous methods have been proposed to solve the control problem in Boolean networks. Among the vast diverse proposed methods, we name the more optimized control techniques, to which a list of possible control nodes is given as input [27–30]. Bo Gao et al proposed an algebraic method to solve the control problem and used the semi-tensor product (STP) as a state transition matrix [31]. Qiu, Yushan et al took benefit from the integer programming to solve the control problem in multiple Boolean networks, for the cancer-causing and normal cells [32]. Christopher James Langmead and Sumit Kumar Jha proposed an algorithm based on model checking to find the control strategy in Boolean networks [33]. Yang Liu et al searches for a controlling sequence to transform from a state to a desirable one, with a difference that he avoids some special and prohibited states [34].

Authors found no exact algorithm for the same network control problem in more recent years. However, an algorithm for identification of the best one-bit perturbation is provided [35]. In this problem, a Boolean network is given and the problem is to find a gene that changing its initial state 1) does not change attractors of the network, 2) maximize the size of the basin of some desired attractors. This method first computes the state graph of the network and then tries to modify the state graph after changing some node’s value, efficiently.

Meanwhile, in some cases, the genetic algorithm and the greedy algorithms are used to solve the control problem in Boolean networks [36–38]. Datta et al proposed an algorithm to control the probabilistic Boolean networks (PBN) based on Markov chains and dynamic programming [24–26]. In this approach, it is supposed that states of some nodes could be controlled externally, and the goal is to find a sequence of changes to be applied as controlling policy to result in the network desirable state. In their method, they first consider the final desired state, and then, they compute a set of desired states for all the time steps in a backward manner. To compute the set of desired states for a time, given set of desired states for the next time step, they enumerate all possible states of the network and their next state. A state with its desired next state is considered as the desired state, for that time step.

Since the Boolean network is a specialization of the probabilistic Boolean network, this method is also applicable to Boolean networks. The problem with the proposed algorithm was that it lacks the necessary efficiency, because all the states within probabilistic Boolean network (or Boolean network) were required to be taken into consideration in all time steps between the initial state and the desirable state; so a state transition matrix with exponential size is produced programmatically [25].

Akutsu et al [39] showed that finding a control strategy for a Boolean network is an NP-hard problem. However, they proposed a polynomial time algorithm to find control strategies over trees, instead of general graphs. The algorithm is based on dynamic programming in which a sub-problem is to find a control sequence for a subtree of the original tree. They also expanded the algorithm for the networks with a low number of loops, but if the network has a high number of loops, or the given number of time steps between the initial state and the desirable state is high, the algorithm would not have the desirable efficiency [39]. Thus, in most of the proposed methods, if the size of the Boolean network is high, the proposed algorithm might have not the desirable efficiency.

Since most biological networks lack a tree structure, the algorithm for tree structures is not applicable for real networks [39]. Therefore, new algorithms are still needed to be more efficient for general networks with a high number of loops, a high number of time steps, and for large size networks. In this paper, we have presented a new algorithm to solve the control problem, and we investigate the applicability of the provided algorithm on two GRNs: T-cell receptor network [40] and Drosophila melanogaster network [41], and compare the efficiency with other algorithms.

In another completely different view, a mathematical formulation of the controllability problem of Boolean networks is provided. These works formulate the dynamics of Boolean networks as linear systems. They defined an extension of matrix multiplication to achieve this goal. They applied this technique on classical Boolean network control problem and showed that this formulation exactly models the desired problem. Le et al proved theorems that specify some necessary and sufficient conditions for a Boolean network to be controllable [42]. The same technique is applied to model a Boolean network with time delays. In a Boolean network with time delay, state of nodes may affect other nodes after some predefined delay. Some necessary and sufficient conditions for controllability of these networks is provided [43]. An extension to the network control problem, which has not only one initial and one desired states, but also a set of states as initial and desired ones (called trajectory), is provided recently [44]. With similar algebraic techniques, they proved theorems on controllability for trajectory problems.

### 1.2 General idea of proposed algorithm

The proposed algorithm is an exact algorithm, i.e. an algorithm that always finds the correct answer, but it is using some properties of the network to enumerate a smaller number of states and perform faster than previously provided algorithms. The general idea of the proposed algorithm is to divide the network to partially-dependent parts and then consider all the possible states the network may be in. To discover these independent subnetworks, we consider strongly connected components and categorize them to find independent parts. Strongly connected components induce an order of dependency between genes. Thus, we can handle them with the induced order, one by one. For each strongly connected component, we consider all possible states. If possible states of a subnetwork (that forms a strongly connected component), is independent of some other parts, we can enumerate these two parts independently, and by this trick, the number of states that we should consider would be reduced. More precisely, if there are *k* independent parts having number of status #*S*_1_, …, #*S*_*k*_, number of status that we consider reduces from #*S*_1_ × … × #*S*_*k*_ to #*S*_1_ + … + #*S*_*k*_. The multiplication is the number of states that the Datta algorithm enumerates. With this trick, we reduce the running time of our algorithm.

As it could be seen, size of strongly connected components of a network could be a good estimation of how faster our algorithm is, in comparison of previously provided dynamic programming algorithms (e.g. Datta algorithm). In a recent study, GRN of E. coli is analyzed. In this network, among 1807 genes, there are 202 transcription factors. This network does not contain any strongly connected component with more than 5 nodes [45]. Also, a manually curated GRN of mouse consists of 274 genes with 176 transcription factors. Its larges strongly connected component contains only 80 genes, in which only 29 are transcription factors [45]. These observations show that real regulatory networks have small strongly connected components which are the cases that our algorithm works better for them. By taking advantages of this observation we provided an algorithm in this paper.

## 2 Materials and methods

### 2.1 Background on Boolean network control

In a Boolean network, each node represents a gene, and each edge represents a regulatory effect of one gene expression on another one, which may cause an increase or decrease in the gene expression [46].

A Boolean network is modelled as a directed graph *G* = (*V*, *F*), which includes a set of *n* nodes *V* = {*v*_1_, *v*_2_, …, *v*_*n*_}, and a set of Boolean functions *F* = {*f*_1_, *f*_2_, …, *f*_*n*_}. Boolean network is a discrete time model. Each node *v*_*i*_ has a state variable *v*_*i*_(*t*) ∈ {0, 1} representing the state of node *v*_*i*_ at time *t*, where 0 (1) indicates lack of (existence of) gene expression. Also, each node *v*_*i*_ has a Boolean function *f*_*i*_, representing how to obtain *v*_*i*_(*t* + 1) from the state of the incoming nodes to *v*_*i*_ at time step *t* by applying basic Boolean operations (**and**, **or**, **not**). The network state at time *t*, is defined as vector *v*^*t*^ = [*v*_1_(*t*), *v*_2_(*t*), …, *v*_*n*_(*t*)], which describes the state of the nodes in time step *t*.

**Fig 1:**
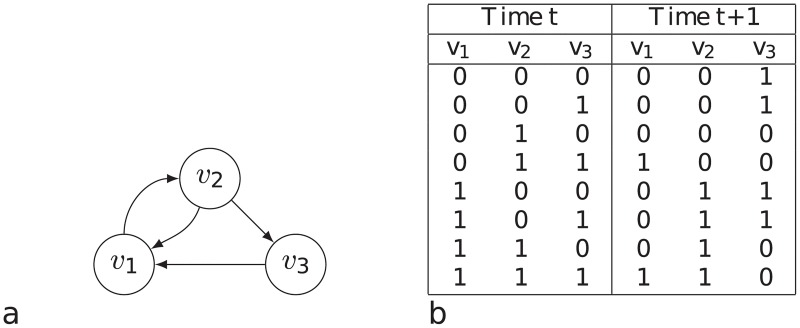
(a) An example of a Boolean network. (b) the state transition table of this Boolean network.

An example of a Boolean network is represented in Fig 1(a). In Fig 1(b), the state transition table of the mentioned Boolean network is represented. This table represents the next state of the network according to the current state. For example, if the network state in time step *t* is [0, 1, 1], then the network state in time step *t* + 1 is [1, 0, 0].

In the control problem of Boolean networks, a Boolean network *G* = (*V*, *F*), initial state and a desirable network state *v*^*τ*^ is given. The set of nodes *V* is called internal nodes. A set of control nodes {*u*_1_, …, *u*_*m*_} are added to the network, known also as external nodes, which are used to influence internal nodes to attain the desirable state. The external nodes have no incoming edges, and their values are specified externally. The problem is to find a sequence of state values *u*^0^, …, *u*^*τ*^ for external nodes, which leads the network to be in desirable state *v*^*τ*^ in time step *τ*. If there exists no such control sequence, this fact should be announced as the output. In gene regulatory networks, the desirable state of the network represents a healthy state of the system, and external nodes represent potential medicines affecting network behavior. Thus, finding control strategies has applications in various medical areas, including medical protocol design for example in cancer treatment [19, 20].

**Fig 2:**
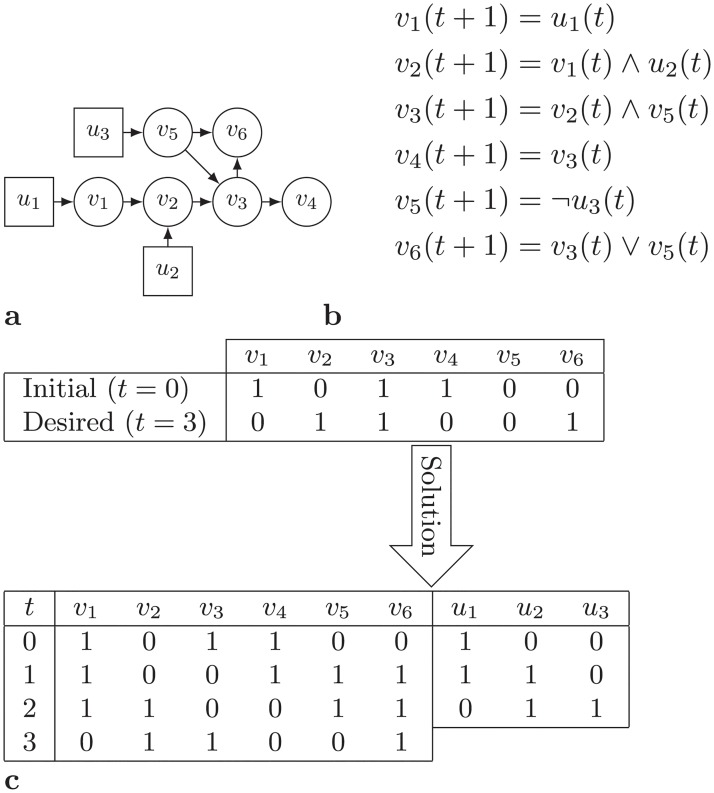
Example of a Boolean network control problem. (a) Network, (b) its state transition rules, (c) Control problem and its solution.

An example of a control problem on a Boolean network is represented in Fig 2. In this example, {*v*_1_, …, *v*_6_} is the set of internal nodes and {*u*_1_, *u*_2_, *u*_3_} is the set of external nodes. The initial state of the network is *v*^0^ = [1, 0, 1, 1, 0, 0], and the desirable state is *v*^3^ = [0, 1, 1, 1, 0, 1]. Thus, we are looking to find a control sequence *u*^0^, …, *u*^3^ in such a way that the network would be in state *v*^3^ at time step *t* = 3. A possible solution, which is shown in the same figure, is *u*^0^ = [1, 0, 0], *u*^1^ = [1, 1, 0], *u*^2^ = [0, 1, 1].

### 2.2 The proposed algorithm

The idea of our proposed algorithm is to reduce backtracking space by separating dependencies and merging information from some sub-parts of the graphs, which we call them components. In addition, we categorize components to find some easily solvable components, non-branching and in some cases the branching components, to reduce the number of states to enumerate.

In our proposed algorithm, we compute an intermediate variable ϒ to be used in the computation of the final result. For each node, at each time step, we calculate answer of two questions: is it possible for this node to be in state 1 (0), and store result of this question in an intermediate variable ϒ. For this computation, we design the following steps.

For network node *v*_*i*_, Boolean variable *b* ∈ {0, 1}, and time step *t*, we define variable ${ϒ}_{b}^{v_{i}}\left(t\right)$, which is *true* if and only if it is possible to assign values to external nodes in such a way that it cause *v*_*i*_(*t*) to get value *b*, and it is *false* otherwise. In the other words, ${ϒ}_{1}^{v_{i}}\left(t\right)$ and ${ϒ}_{0}^{v_{i}}\left(t\right)$ represent the possibility for node *v*_*i*_ in time step *t* to have state value 1 and 0, respectively.

Let $v_{i_{1}}\ldots v_{i_{k}}$ be the input nodes to node *v*_*i*_. According to the definition of ϒ we have ${ϒ}_{1}^{v_{i}}\left(t+1\right)=true$ if, and only if, there exists $\left[b_{i_{1}},b_{i_{2}},\ldots ,b_{i_{k}}\right]$ such that $f_{i}\left(b_{i_{1}},b_{i_{2}},\ldots ,b_{i_{k}}\right)=1$ and ${ϒ}_{b_{i_{j}}}^{v_{i_{j}}}\left(t\right)=true$ for all *j* = 0, …, *k*. Value of ${ϒ}_{0}^{v_{i}}\left(t+1\right)=true$, could be computed in the same way.

For example, for node *v*_6_ in Fig 2, ${ϒ}_{1}^{v_{6}}\left(t+1\right)$ is true if at least one of the variables ${ϒ}_{1}^{v_{3}}\left(t\right)$ and ${ϒ}_{1}^{v_{5}}\left(t\right)$ are true. On the other hand, variable ${ϒ}_{0}^{v_{6}}\left(t+1\right)$ is true if both variables ${ϒ}_{0}^{v_{3}}\left(t\right)$ and ${ϒ}_{0}^{v_{5}}\left(t\right)$ are true.

For constant node *v*_*i*_, either ${ϒ}_{1}^{v_{i}}\left(t\right)=true$ and ${ϒ}_{0}^{v_{i}}\left(t\right)=false$ or ${ϒ}_{1}^{v_{i}}\left(t\right)=false$ and ${ϒ}_{0}^{v_{i}}\left(t\right)=true$ are true for all time steps. Also, for each external node *u*_*i*_, both ${ϒ}_{1}^{u_{i}}\left(t\right)=true$ and ${ϒ}_{0}^{u_{i}}\left(t\right)=true$ are true for all time steps.

To obtain the final result, we should check the existence of a control sequence which leads to ${ϒ}_{v^{\tau }\left[i\right]}^{v_{i}}\left(\tau \right)=true$ for all the nodes. Also, to specify and output the desired control sequence, the regression technique may be used [39].

**Fig 3:**
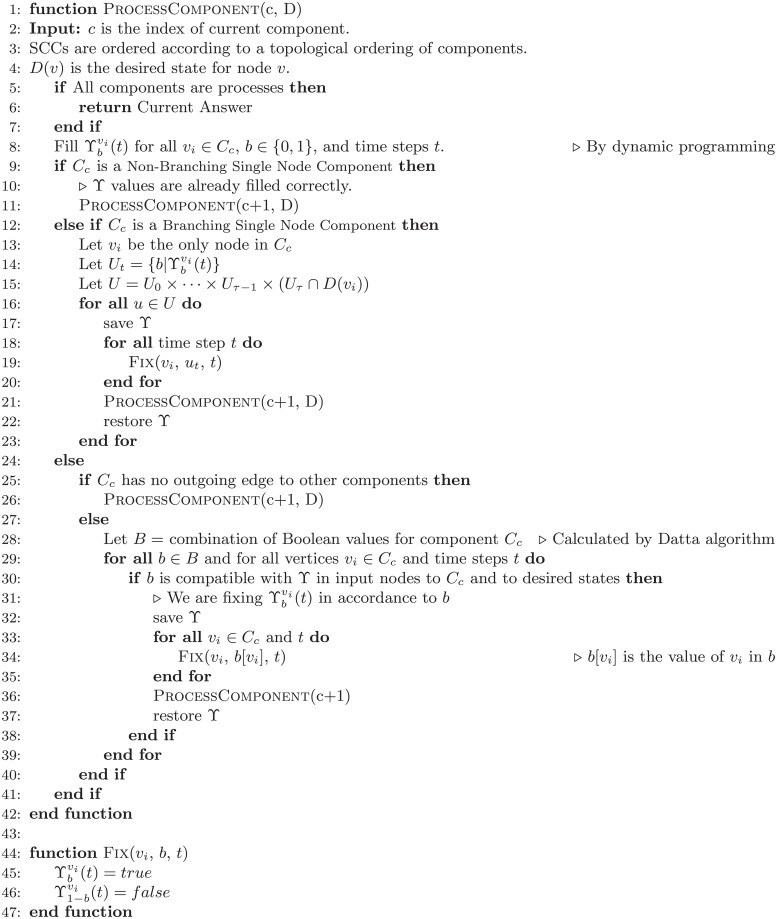
Pseudo-code of our proposed algorithm.

Pseudocode of our proposed algorithm is presented in Fig 3. The source codes are available as S1 Codes.

#### 2.2.1 Strongly connected components

In order to compute ϒ values, we partition the network into strongly connected components.

**A strongly connected component** (SCC) of a network, is a maximal subset of network nodes, in which every node is reachable from every other one. We partition the network nodes into strongly connected components, using SCC algorithm [47]. This algorithm is one of the classical graph theory algorithms based on two depth-first searches on graph and reverse of the graph, respectively. **A topological order** on strongly connected components of a network, is an order on its components such that for every directed edge *xy* from component *x* to component *y*, *x* appears before *y* in the ordering.

We find a topological order on the components using topological sort algorithm [47]. Then, based on the size of the component and the number of outputs edges, we categorize components into three categories: 1) *non-branching single node components*, 2) *branching single node components*, 3) *multi-node components*. A branching node is a node with at least two outgoing edges and a non-branching node is a node with at most one outgoing edge.

#### 2.2.2 Non-branching single node components

In this case, current component consists of one node *v*_*i*_, and *v*_*i*_ has at most one outgoing edge. We simply find ${ϒ}_{0}^{v_{i}}\left(t\right)$ and ${ϒ}_{1}^{v_{i}}\left(t\right)$ for 0 ≤ *t* ≤ *τ* from state values of incoming nodes to *v*_*i*_ in last time step.

**Fig 4:**
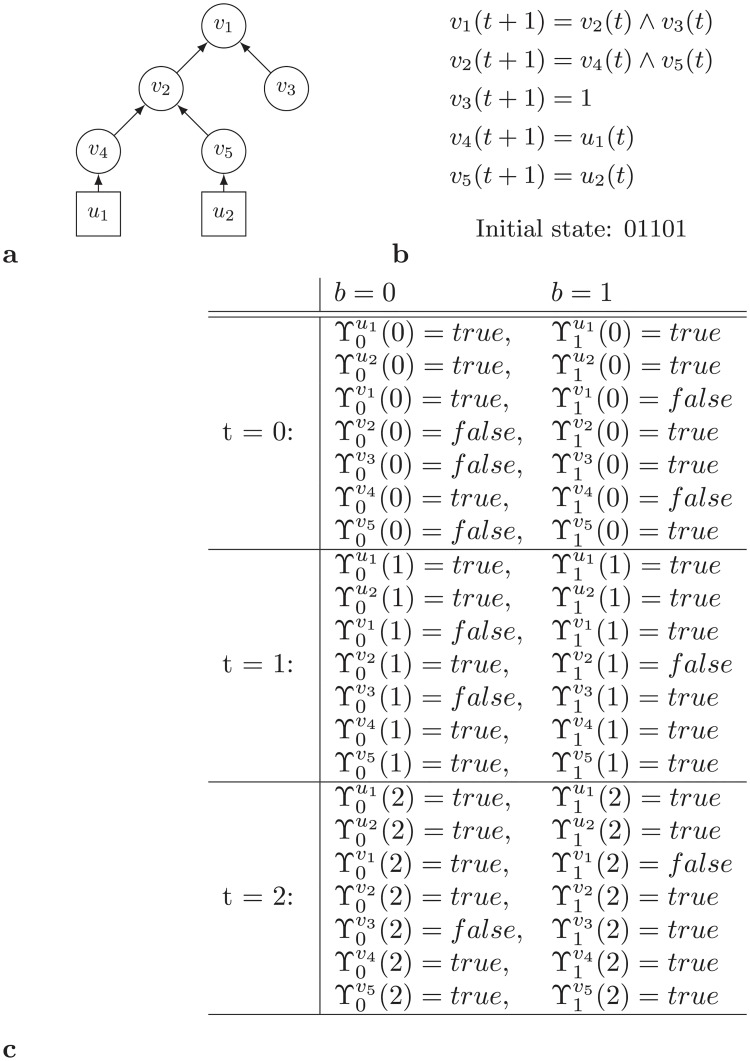
Example of a network with four non-branching single node components.

For example, node *v*_2_ in Fig 2 is a Non-Branching Single Node Component for which ${ϒ}_{0}^{v_{2}}\left(t\right)$ and ${ϒ}_{1}^{v_{2}}\left(t\right)$ for 0 ≤ *t* ≤ 2 should be calculated based on state values of their incoming nodes *v*_1_ and *u*_2_ in the previous time step. Note that, interestingly, for node *v*_2_ in time step *t* = 2 it is possible to be in state 1 and state 0. Another example with four non-branching single node components and its ϒ values is present in Fig 4.

In accordance with dynamic programming arrays, we fill ϒ to desirable time step *τ*. Since the nodes are met based on a topological sort, and all the states of the incoming nodes of the related node till the time step *τ* are calculated before, this calculation is possible. In time step *τ*, check if the related node has attained the desirable state or not. If the answer was negative, announce that there is no control sequence.

#### 2.2.3 Branching single node components

**Fig 5:**
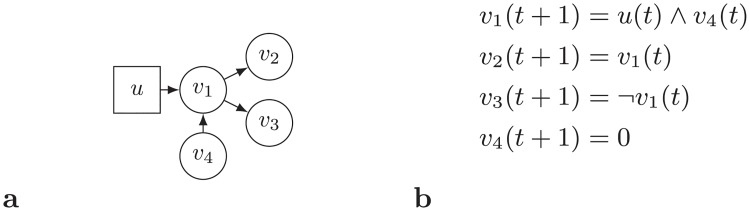
An example of a branching node that is not able to attain both states 0 and 1 in a time step.

In this case, current component consists of one node *v*_*i*_, and *v*_*i*_ has at least two outgoing edges. Same as the previous case, we can simply find values ${ϒ}_{0}^{v_{i}}\left(t\right)$ and ${ϒ}_{1}^{v_{i}}\left(t\right)$. The difference is that since *v*_*i*_ has more than two outgoing edges, there are at least two other nodes in the network which their state values depend on the state value of *v*_*i*_ in the previous time. Thus, in some networks, the state value of *v*_*i*_ which is required by its outgoing neighbors may be inconsistent. An example of a branching node that is not able to attain both states 0 and 1 in a time step is present in Fig 5.

Suppose that initial state is 101100 (as it is the case in Fig 2) and we are supposed to reach to a state with *v*_6_ = 0 and *v*_3_ = 1 in time step *t* = 4. If we treat this node same as Non-Branching Single Node Components, we would face a problem. This algorithm announces that in time step *t* = 4, we can have *v*_6_ = 0 and *v*_3_ = 1, but there is no control sequence reaches to a state with *v*_6_ = 0 and *v*_3_ = 1 in *t* = 4. The problem is that both *v*_3_ and *v*_5_ in *t* = 3 are able to attain values 0 and 1, but, there is a dependency between them. This dependency does not let *v*_6_ = 0 and *v*_3_ = 1 in *t* = 4, simultaniously.

To deal with this problem, we fix some value for the node and recursively consider nodes for next SCCs. In this manner, we remove the dependency between the following nodes by considering different states one by one, recursively.

In other words, as we detect a dependency between states, we enumerate recursively all possible states. In the case of the branching node, if the branching node has more than one state in time *t*, we go through backtracking. Otherwise, we can branch-out some states. If the node has one possible state, there is no need to go through backtracking. We can simply fill the next components sequentially.

#### 2.2.4 Multi-node components

In this case, the current component consists of at least two nodes. We apply Datta algorithm in this case [24]. Datta et al fill an array *D*[*v*_1_(*t*), *v*_2_(*t*), …, *v*_*n*_(*t*), *t*], for *t* = *τ* to *t* = 0 according to the following procedure:

$$D\left[v_{1}(t),v_{2}(t),\ldots ,v_{n}(t),\tau \right]=\{\begin{array}{ll} 1, & \text{if}\;\left[v_{1}(t),v_{2}(t),\ldots ,v_{n}(t)\right]=v^{\tau }\\ 0, & \text{otherwise} \end{array}$$ (1)

$$D\left[v_{1}\left(t^{\prime }\right),v_{2}\left(t^{\prime }\right),\ldots ,v_{n}\left(t^{\prime }\right),t^{\prime }\right]=\{\begin{array}{l} 1,\begin{array}{l} \;\text{if}\;\text{there}\;\text{exists}\;\left(v^{t},u\right)\text{such}\;\text{that}\\ \;D\left[v_{1}(t),v_{2}(t),\ldots ,v_{n}(t),t\right]=1\;\text{and}\\ \;v^{t}=f\left(v^{t^{\prime }},u\right) \end{array}\\ 0,\begin{array}{l} \;\text{otherwise} \end{array} \end{array}$$ (2)

where *t*′ = *t* − 1. The table *D*[*v*_1_, …, *v*_*n*_, *t*] shows the possibility of reaching to the desired state, if we start from state *v*_1_, …, *v*_*n*_ from time step *t*. Thus, there exists a desired solution if and only if *D*[*v*_1_(0), *v*_2_(0), …, *v*_*n*_(0), 0] = 1 holds for *v*_1_(0), …, *v*_*n*_(0).

After applying Datta algorithm, we check if it is possible to attain the desirable state in time step *t* = *τ*. Then, we fill ϒ array accordingly. For example, node *v*_*i*_ in time step *t* may attain the desired state in time step *τ* with both states 0 and 1, thus, we will have ${ϒ}_{0}^{v_{i}}\left(t\right)=true$ and ${ϒ}_{1}^{v_{i}}\left(t\right)=true$. Then we partition nodes with edges going out of the component into two categories, nodes with at most one outgoing edge and nodes with at least two outgoing edges (branching node). Nodes with at most one outgoing edge are easy to handle, however, we treat branching nodes which may attain both states 0 and 1 in the same time step, like *branching single node components*. In both cases, we fix values for this component and go for the next component recursively.

#### 2.2.5 Complexity analysis

Let *C*_1_, …, *C*_#*C*_ be the strongly connected components of *G*, and *σ*_*t*_(*C*_*i*_) be the number of valid states of component *C*_*i*_ at time step *t*. Our algorithm enumerates all the network states, but non-branching single node components and branching single node components.

Datta algorithm finds all potentially desirable network states backward in time. To find all desirable states in time step *t*, it generates all potential states for the network, which takes *O*(2^*n*^) time, for an *n* node network. Then, for each potential state, it calculates next state and checks whether it leads to the desirable state by searching it within desirable states of the time step *t* + 1. Let *T*(*G*) be the time required to calculate the next state of a network’s state. The amount of computation for time step *t*, for each potential state is *T*(*G*) for calculating the next step in addition to searching it, which takes lg *σ*_*t*+1_(*G*). Datta performs these two operations for all 2^*n*^ potential states. Therefore, Datta algorithm’s running time is *O*(∑_*t*_ 2^*n*^(*T*(*G*) + lg *σ*_*t*_(*G*))) = *O*(*τ*2^*n*^*T*(*G*)). Note that, calculation of *T*(*G*) requires computation of Boolean functions for each node, and if functions are given as input, their calculation asymptotic time is not more than the size of the input, which is acceptable.

On the other hand, our algorithm proceeds component by component. For each component, we consider all the *σ*_*t*_(*C*_*i*_) states for all time steps *t*. However, in the following three cases, we do not enumerate all the states:

- For non-branching single node components, we compress states. In other words, we check further components not by considering two possible states for these components recursively. However, since network states of following components do not depend on different values of these components, we make our enumerations by half, for each of these components. Thus, if we have *k* such components, our running time will be reduced by a factor of $\frac{1}{2^{k}}$, in comparison to normal dynamic programming.
- We can check produced network states to be compatible with desired states when considering a component, and we do not delay it until enumerating all the following possible states. We cleverly do this, by not backtracking on the time step *t* = *M*, but only on time steps *t* = 1 through *M* − 1. Since no further network state depends on time step *t* = *M* of any component, we may only check this compatibility and not backtracking on them.
- Leaf components with out-degree 0 are independent, and not required to be checked recursively, but sequentially.

Considering above notes, our running time is *O*(Σ_*t*_Π_*i*:*I*(*t*)_*σ*_*t*_(*C*_*i*_)*T*(*C*_*i*_)). Note that since 2^*n*^ ≥ *σ*_*t*_(*G*) ≥ *Π*_*i*_*σ*_*t*_(*C*_*i*_) and *T*(*G*) ≥ ∑_*i*_ *T*(*C*_*i*_), our running time is always better than running time of dynamic programming.

### 2.3 Example

**Fig 6:**
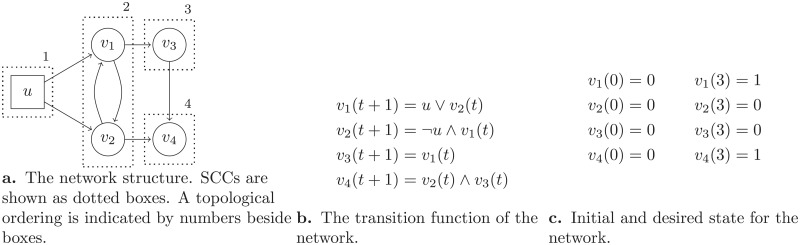
Example of a network, its transition function, and its initial and desired states.

Consider the network shown in Fig 6. This network consists of one external node *u* and four internal nodes *v*_1_, *v*_2_, *v*_3_, and *v*_4_. Transition function and initial/desired state for the network is shown in Fig 6.

Based on the proposed algorithm, we first partition network nodes to SCCs. SCCs of the network of Fig 6 are *S*_1_ = {*u*}, *S*_2_ = {*v*_1_, *v*_2_}, *S*_3_ = {*v*_3_} and *S*_4_ = {*v*_4_}. SCCs are shown in Fig 6 as dotted boxes.

First we process SCC *S*_1_ = {*u*}. Since node *u* is an external node, in every time step *t*, *u* can attain both states 0 and 1, thus, ${ϒ}_{b}^{u}\left(t\right)=true$ for all 0 ≤ *t* ≤ 3 and *b* ∈ {0, 1}. This SCC is a branching single node component. Thus, we fix ${ϒ}_{b}^{u}\left(t\right)$ for all 0 ≤ *t* ≤ 3 for all different combinations of assignment values, as it is represented in Table 1. In the recursion, if correct solution is found, the algorithm reports the solution and halts. Otherwise, the algorithm fixes another combination and goes for next SCC, recursively. For this example, suppose that the algorithm fixes row #2 of the Table 1 for component *S*_1_, and then goes through next SCCs.

**Table 1:** State space for the SCC *S*_1_ for the network represented in Fig 6.

	t = 0	t = 1	t = 2	t = 3
#1	0	0	0	0
#2	1	0	0	0
#3	0	1	0	0
#4	1	1	0	0
#5	0	0	1	0
#6	1	0	1	0
#7	0	1	1	0
#8	1	1	1	0
#9	0	0	0	1
#10	1	0	0	1
#11	0	1	0	1
#12	1	1	0	1
#13	0	0	1	1
#14	1	0	1	1
#15	0	1	1	1
#16	1	1	1	1

Next SCC, according to the topological order of SCCs, is *S*_2_ = {*v*_1_, *v*_2_}. The logic behind behavior of *S*_2_ is that, if *u*(*t*) = 1, then, we have *v*_1_(*t* + 1) = 1 and *v*_2_(*t* + 1) = 0, and if *u* = 0, *v*_1_ and *v*_2_ exchange their state at the next time step. *S*_2_ is a multi-node component. Thus, the algorithm first executes a Datta algorithm on this SCC (line 28 of Fig 3). The result of Datta algorithm is shown in Table 2. As it is shown in Table 2, since we already fixed one state over all time steps for SCC *S*_1_, there is only one possible state for the *S*_2_. For SCC *S*_2_, after calculating ϒ table, since *S*_2_ is a multi-node component, the algorithm fixes this state and goes through next SCCs.

**Table 2:** Value of ϒ table for SCC *S*_2_ for the network represented in Fig 6.

		t = 0	t = 1	t = 2	t = 3
*v*_1_	b = 0	${ϒ}_{0}^{v_{1}}\left(t\right)=\text{true}$	${ϒ}_{0}^{v_{1}}\left(t\right)=\text{false}$	${ϒ}_{0}^{v_{1}}\left(t\right)=\text{true}$	${ϒ}_{0}^{v_{1}}\left(t\right)=\text{false}$
	b = 1	${ϒ}_{1}^{v_{1}}\left(t\right)=\text{false}$	${ϒ}_{1}^{v_{1}}\left(t\right)=\text{true}$	${ϒ}_{1}^{v_{1}}\left(t\right)=\text{false}$	${ϒ}_{1}^{v_{1}}\left(t\right)=\text{true}$
*v*_2_	b = 0	${ϒ}_{0}^{v_{2}}\left(t\right)=\text{true}$	${ϒ}_{0}^{v_{2}}\left(t\right)=\text{true}$	${ϒ}_{0}^{v_{2}}\left(t\right)=\text{false}$	${ϒ}_{0}^{v_{2}}\left(t\right)=\text{true}$
	b = 1	${ϒ}_{1}^{v_{2}}\left(t\right)=\text{false}$	${ϒ}_{1}^{v_{2}}\left(t\right)=\text{false}$	${ϒ}_{1}^{v_{2}}\left(t\right)=\text{true}$	${ϒ}_{1}^{v_{2}}\left(t\right)=\text{false}$

Next SCC, *S*_3_ = {*v*_3_}, is a single node non-branching component. Thus, the algorithm calculates ${ϒ}_{b}^{v_{3}}\left(t\right)$ for all 1 ≤ *t* ≤ 3 and *b* ∈ {0, 1}. By the definition of transition function (Fig 6), ${ϒ}_{b}^{v_{3}}\left(t+1\right)={ϒ}_{b}^{v_{1}}\left(t\right)$. Then, we proceed to next SCC.

Next SCC is *S*_4_ = {*v*_4_}. So far, the value of ϒ table is shown in Table 3. Like SCC *S*_3_, SCC *S*_4_ is a single node non-branching component. Thus, the algorithm first calculates ϒ table. According to the transition function (Fig 6), ${ϒ}_{1}^{v_{4}}\left(t+1\right)={ϒ}_{1}^{v_{2}}\left(t\right)\wedge {ϒ}_{1}^{v_{3}}\left(t\right)$ and ${ϒ}_{0}^{v_{4}}\left(t+1\right)={ϒ}_{0}^{v_{2}}\left(t\right)\vee {ϒ}_{0}^{v_{3}}\left(t\right)$. Now, the algorithm processed all the SCCs, thus, it reports the result.

**Table 3:** Value of ϒ table after processing SCCs *S*_1_, *S*_2_, and *S*_3_ and before processing SCC *S*_4_ for the network represented in Fig 6.

		t = 0	t = 1	t = 2	t = 3
*u*	b = 0	${ϒ}_{0}^{u}\left(t\right)=\text{false}$	${ϒ}_{0}^{u}\left(t\right)=\text{true}$	${ϒ}_{0}^{u}\left(t\right)=\text{true}$	${ϒ}_{0}^{u}\left(t\right)=\text{true}$
	b = 1	${ϒ}_{1}^{u}\left(t\right)=\text{true}$	${ϒ}_{1}^{u}\left(t\right)=\text{false}$	${ϒ}_{1}^{u}\left(t\right)=\text{false}$	${ϒ}_{1}^{u}\left(t\right)=\text{false}$
*v*_1_	b = 0	${ϒ}_{0}^{v_{1}}\left(t\right)=\text{true}$	${ϒ}_{0}^{v_{1}}\left(t\right)=\text{false}$	${ϒ}_{0}^{v_{1}}\left(t\right)=\text{true}$	${ϒ}_{0}^{v_{1}}\left(t\right)=\text{false}$
	b = 1	${ϒ}_{1}^{v_{1}}\left(t\right)=\text{false}$	${ϒ}_{1}^{v_{1}}\left(t\right)=\text{true}$	${ϒ}_{1}^{v_{1}}\left(t\right)=\text{false}$	${ϒ}_{1}^{v_{1}}\left(t\right)=\text{true}$
*v*_2_	b = 0	${ϒ}_{0}^{v_{2}}\left(t\right)=\text{true}$	${ϒ}_{0}^{v_{2}}\left(t\right)=\text{true}$	${ϒ}_{0}^{v_{2}}\left(t\right)=\text{false}$	${ϒ}_{0}^{v_{2}}\left(t\right)=\text{true}$
	b = 1	${ϒ}_{1}^{v_{2}}\left(t\right)=\text{false}$	${ϒ}_{1}^{v_{2}}\left(t\right)=\text{false}$	${ϒ}_{1}^{v_{2}}\left(t\right)=\text{true}$	${ϒ}_{1}^{v_{2}}\left(t\right)=\text{false}$
*v*_3_	b = 0	${ϒ}_{0}^{v_{3}}\left(t\right)=\text{true}$	${ϒ}_{0}^{v_{3}}\left(t\right)=\text{true}$	${ϒ}_{0}^{v_{3}}\left(t\right)=\text{false}$	${ϒ}_{0}^{v_{3}}\left(t\right)=\text{true}$
	b = 1	${ϒ}_{1}^{v_{3}}\left(t\right)=\text{false}$	${ϒ}_{1}^{v_{3}}\left(t\right)=\text{false}$	${ϒ}_{1}^{v_{3}}\left(t\right)=\text{true}$	${ϒ}_{1}^{v_{3}}\left(t\right)=\text{false}$

Note that, in any of the recursion steps, if the algorithm does not find an answer that matches to the desired state, it traces back to the SCCs that are not single node non-branching, tries the next state from the state space. In the case of the current example, the algorithm will traceback for the next state for *S*_2_, which does not have any other, and then traces back to *S*_1_ for the next state.

### 2.4 Proof of the correctness of the proposed algorithm

In a general view, the proposed algorithm enumerates all possible states, but, removes some useless states. There are two extreme approaches to enumerate the states. One approach is to recursively enumerate them node-by-node. The other extreme, like Datta algorithm, is to fill an array that represents reachability of the states, time step-by-time step. Our algorithm is somewhat in-between, it fills arrays for SCCs, and fix them recursively.

We provide a stronger result for the proposed algorithm. We let logical formulas that describe states of the nodes in time step *t* + 1, to not only include variables of the time step *t*, but also variables of time steps 0 to *t*. We name these networks as extended networks. Also, we name single node non-branching SCCs as simple and the other SCCs as hard components.

**Fig 7:**
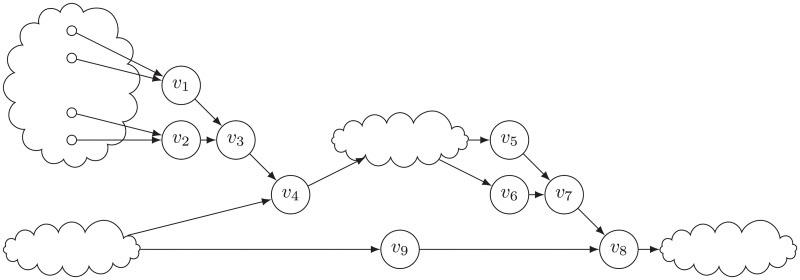
A schematic view of the topology of single node non-branching components between other SCCs of a network. Cloudy nodes are either branching single node or multi-node components (hard components). Normal circles represent single node non-branching components (simple components). Simple components make a rooted tree toward their roots.

Consider all the non-branching single node components that proceed an SCC *S* in the topological order. All easy components may have 0 or more input edges, but only one output edge. Note that, if there is a single node non-branching SCC without output edge, it could be removed from the network. After finishing the process of the network, these nodes could be checked for its consistency with the desired state at the end. These simple component nodes make a rooted tree which is directed toward the root. A schematic view is shown in Fig 7.

**Fig 8:**
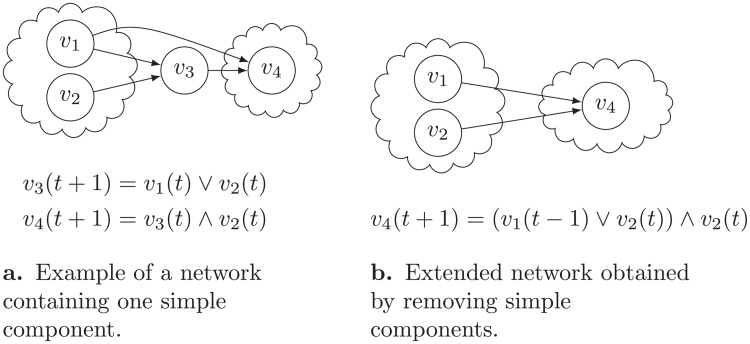
Example of applying the transformation of removing simple components from a network to obtain a simple component-free extended network.

We can replace them in all logical formulas by the state of their input nodes in earlier time steps, or in some cases by nodes’ initial states. As an example, consider the schematic view of the network shown in Fig 8. In this network, we can replace *v*_3_ in all transition formulas by its inputs and change the network accordingly, as it is shown in Fig 8. The resulting network is a simple component-free extended network.

By this technique, we will have an extended network without any simple SCC. We provide a proof of correctness of our algorithm on these extended networks. While we proved the correctness of the provided algorithm, on these extended networks, as a direct result, the correctness of our algorithm on hard components follows immediately. Also, it is shown that simple components are derived deterministically from the state of nodes within hard components. This shows the correctness of the proposed algorithm.

Considering a simple component-free extended network, the proposed algorithm processes it SCC by SCC. The algorithm first generates all possible states for each SCC, and then enumerates them one by one and goes for the next SCC, recursively. In other words, instead of enumerating all the states for the network node by node, it makes groups of nodes (SCCs) and enumerates states for them. It is clear in this case that, the algorithm enumerates states just like a brute force algorithm. Since the brute force algorithm enumerates and checks all the states, its correctness is obvious from its definition. Note that, the proposed algorithm is not just one brute force, but, we reduced the correctness proof of our algorithm to the correctness of a brute force algorithm.

## 3 Application to biomolecular network control

### 3.1 Drosophila Melanogaster’s network

**Fig 9:**
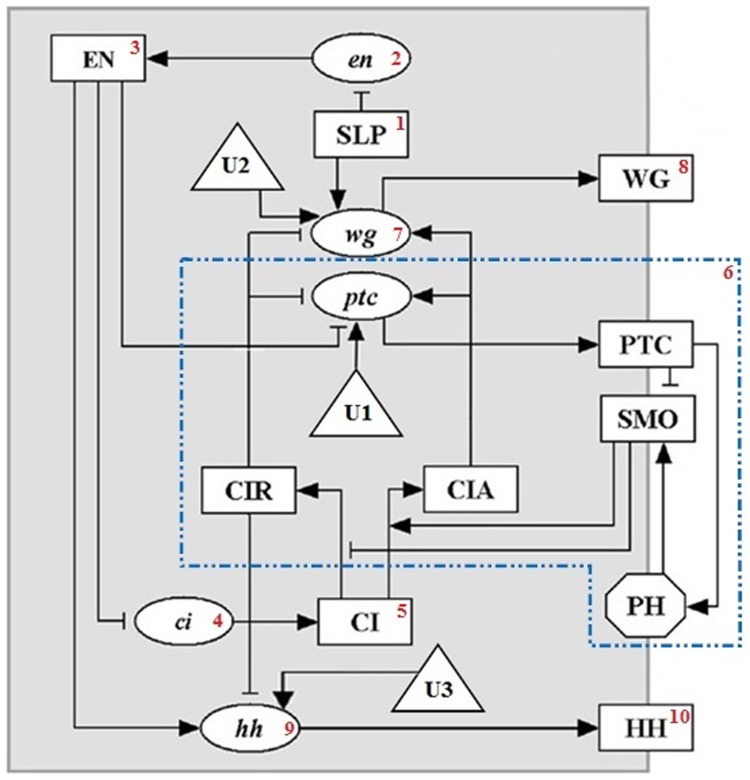
Boolean network model of Drosophila melanogaster. Dotted lines indicate a *multi-node component*, and the numberings beside nodes indicate topological orders. Nodes indicated as rectangles, i.e. nodes U1, U2, and U3, are external nodes.

In this section, we present the steps of applying the proposed algorithm on Drosophila melanogaster subnetwork [41], which contains 15 nodes. As it can be seen in Fig 9, three external nodes U1, U2 and U3 are added to this network. Evolving functions regarding the added control sequences are shown in Table 4.

**Table 4:** Evolution functions for the Boolean network model of Drosophila melanogaster.

Node	Boolean updating function
SLP	SLP^*t*+1^ = SLP^*t*^
wg	wg^*t*+1^ = ((CIA^*t*^ ∧ SLP^*t*^ ∧ ¬CIR^*t*^) ∨(wg^*t*^ ∧ (CIA^*t*^ ∨ SLP^*t*^) ∧ ¬CIR^*t*^)) ∧ U2^*t*^
WG	WG^*t*+1^ = wg^*t*^
en	en^*t*+1^ = ¬SLP^*t*^
EN	EN^*t*+1^ = en^*t*^
hh	hh^*t*+1^ = EN^*t*^ ∧ ¬CIR^*t*^ ∧ U3^*t*^
HH	HH^*t*+1^ = hh^*t*^
ptc	ptc^*t*+1^ = CIA^*t*^ ∧ ¬EN^*t*^ ∧ ¬CIR^*t*^ ∧ U1^*t*^
PTC	PTC^*t*+1^ = ptc^*t*^ ∧ PTC^*t*^
PH	PH^*t*+1^ = PTC^*t*^
SMO	SMO^*t*+1^ = ¬PTC^*t*^
ci	ci*ci*^*t*+1^ = ¬EN^*t*^
CI	CI^*t*+1^ = ci^*t*^
CIA	CIA^*t*+1^ = CI^*t*^ ∧ SMO^*t*^
CIR	CIR^*t*+1^ = CI^*t*^ ∧ ¬SMO^*t*^

First, we partitioned the network’s graph into strongly connected components. As it could be seen in Fig 9, graph has only one *multi-node component*, which is shown through dotted lines. Then, nodes are ordered based on topological sort and the result is shown as numberings beside each node in Fig 9.

We treat graph nodes one by one according to their topological ordering. First, “SLP” node is met. This node is a *none-branching single node component*; therefore, we compute ϒ for this node till *t* = *τ*. Since this node is a constant node (a node without entering edge), it will always keep its initial state. The ϒ concerning this node will be filled to this constant value. Then, for time step *τ*, we check that if this node has attained the desirable state or not. If not, it would be announced that there exist no desired control sequence, and the algorithm terminates.

Next, the second node in topological sort that is the “en” node will be visited. This node is also a *none-branching single node component*, thus, it is processed the same as “SLP” node.

Then the “EN” node is considered. It is a branching node, but since it has no control node before itself, it can attain only one state in every time step. Therefore this node is treated like “SLP” and “EN” nodes. For “ci” and “CI” nodes, the very procedure will go.

Now we reach a *multi-node component*. For this strongly connected component, the dynamic programming is computed, then it is checked if there is a possibility to attain the desired state in time step *τ* for the nodes inside this component or not. If we can attain the desirable state within the time step *τ*, the ϒ for each node would be filled. Then, branching nodes which have outgoing edges from the component, would be handled as “CIR” node to check if these nodes are possible to attain two states of 0 and 1 in a time step. If they could, a state sequence that we can attain the desirable state, considered and the algorithm would be recalled for the network’s remaining nodes.

Then nodes “wg”, “WG”, “hh” and “HH” are considered. these nodes are *none-branching single node components*. Note that, if for example “CIR” with two state sequences can attain the desirable state, and for the first state sequence, it would not be possible for the next nodes (e.g. “wg”) to attain the desirable state, the algorithm will return and for the sequences of the second state of “CIR”, it would check the remaining nodes. In the case that all the nodes were possible to attain the desirable state, it will be announced that there exists a control sequence that causes attaining the desirable state in time step *τ*.

### 3.2 T-Cell receptor kinetics’ network

**Fig 10:**
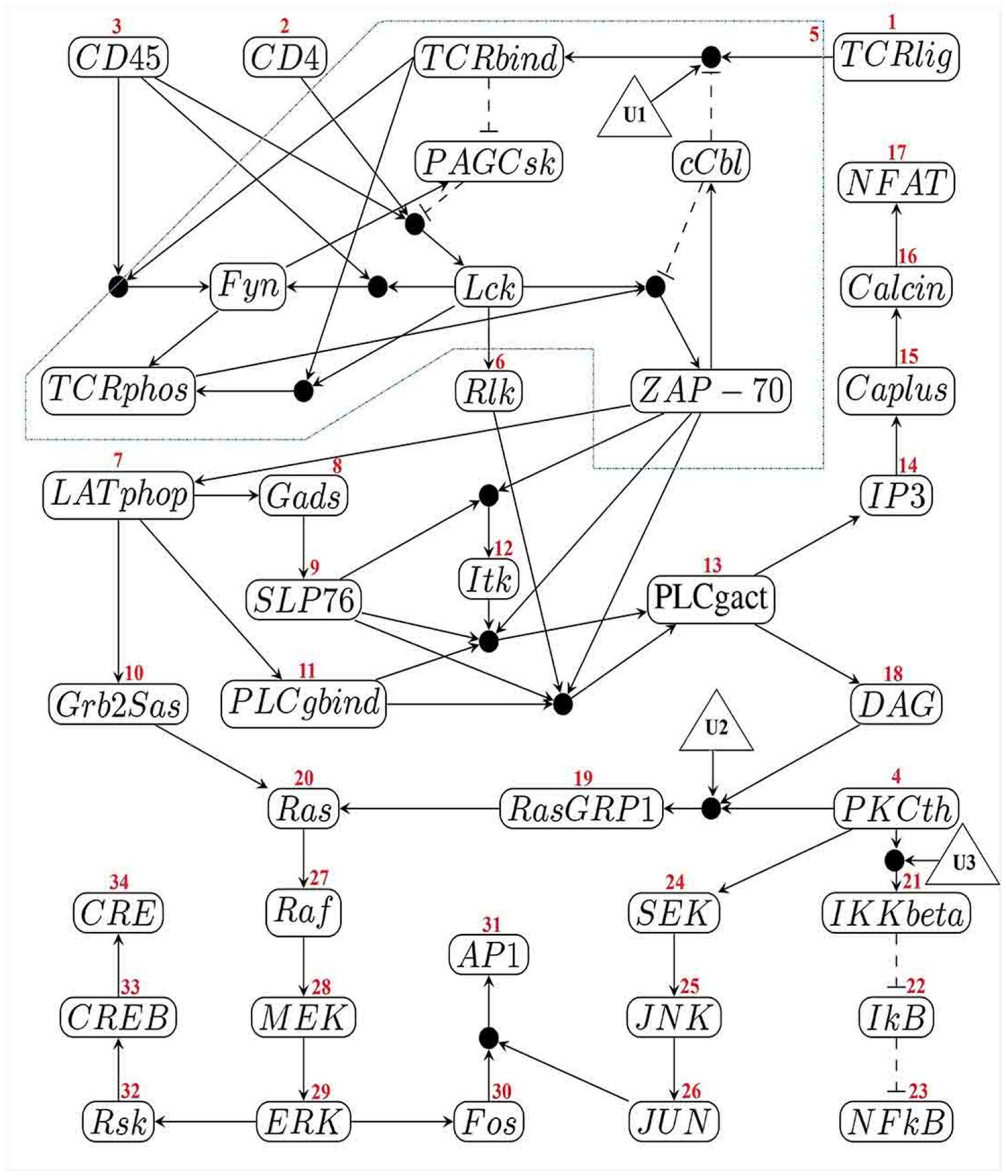
Boolean network model of T-cell receptor. Network’s graph has one *multi-node component*. The numberings beside each node indicate their topological ordering.

We apply the proposed algorithm to the Boolean network model of T-cell receptor kinetics [40]. The *multi-node component* and the order of nodes based on a topological sort are depicted in Fig 10. As it is obvious in this figure, this model of the Boolean network has 40 genes and one *multi-node component*. Three external nodes U1, U2, and U3, are added to the Boolean network.

In T-cell receptor kinetics, the network which can be seen in Fig 10, arrows with pointed heads represent activation and dashed arrows with bar heads represent inhibition (the dashed arrows represent “not” which is related to that node). The network is represented as a network of “or”s of “and”s. Thus, large filled circles representing “and” of their inputs, while when edges are going to a node, state of the node is determined according to the “or” of its incoming edges. For example

$$\begin{array}{c} \text{Fyn}(t+1)=(\text{CD45}(t)\wedge \text{TCRbind}(t))\vee (\text{CD45}(t)\wedge \text{Lck}(t)) \end{array}$$

Our suggested algorithm on the Boolean network model of Drosophila melanogaster was implemented for an initial state (in time step *t* = 0) and a desired state (in time step *t* = 6), which is depicted in Table 5. It is noteworthy to say that there exists a control sequence to reach the desirable state in time step *t* = 6. Also, the Datta et al algorithm was implemented in this dataset, for comparison. Note that, since this dataset has 22 edges and 15 internal nodes, the algorithm presented by Akutsu et al requires a tree which has 8 fewer edges from this network (*H* = 8).

**Table 5:** Initial state and desirable state of each node for Drosophila melanogaster network’s graph.

Node	Initial State (*t* = 0)	Desired State (*t* = 6)
en	0	1
EN	0	1
SLP	0	0
wg	1	0
WG	1	0
ptc	0	0
PTC	0	0
CIA	0	0
CIR	1	0
ci	0	0
CI	1	0
SMO	0	1
PH	0	0
hh	1	0
HH	0	1

### 3.3 Comparison with previous methods

With initial and desired states that are mentioned in Table 6, we evaluated the proposed algorithm on the Boolean network of Drosophila melanogaster and compared it with previous algorithms. This dataset contains 22 edges and 15 internal nodes, the algorithm presented by Akutsu et al requires a tree which has 8 fewer edges from this network. Thus all combinations of 8 edges are to be removed from the graph that makes it very slow for this case.

**Table 6:** Initial states and desirable states of each node in T-cell receptor kinetics network graph.

Node	Initial State (*t* = 0)	Desired State (*t* = 5)	Node	Initial State (*t* = 0)	Desired State (*t* = 5)
CD45	1	1	Grb2Sas	0	0
CD4	0	0	PLCgbind	0	0
TCRbind	0	1	DAG	0	0
TCRlig	1	1	Ras	0	0
PAGCsk	0	1	RasGRP1	0	0
cCbl	0	0	PKCth	1	1
NFAT	0	0	CRE	0	0
Fyn	0	1	Raf	0	0
Lck	1	0	SEK	0	1
Calcin	0	0	IKKbeta	0	1
TCRphos	0	1	CREB	1	0
Rlk	0	0	MEK	0	1
ZAP-70	0	0	AP1	0	0
Caplus	0	0	JNK	0	1
LATphop	0	0	IKB	1	0
Gads	0	0	Rsk	0	0
IP3	0	0	ERK	0	0
SLP76	0	0	Fos	1	0
Itk	0	0	JUN	0	1
PLCgact	1	0	NFkB	0	1

The initial state and desired state for T-cell receptor model is shown in Fig 10. This dataset has 40 internal nodes (*n* = 40) and 3 external nodes (*m* = 3). We compared the proposed algorithm with the algorithm of Datta et al and Akutsu et al on these initial and desired conditions. Note that there is a control sequence for the above mentioned desirable state in time step *t* = 5.

Akutsu et al [39] first provide a polynomial time algorithm for directed trees. However, their algorithm does not work on general graphs. For general graphs, they remove some edges from the graph to obtain a tree and then search for all possible solutions. Among all the solutions, if one is compatible with the removed edges, their algorithm reports the solution. Note that, since they have to obtain all solutions for the obtained trees, their algorithm on general graphs is not a polynomial time algorithm anymore.

The algorithm presented by Akutsu et al requires a tree which has 19 fewer edges from this network (*H* = 19). Time complexity of this algorithm is *O*(2^*H*(*τ*+1)^(*n* + *m*)*τ*), which is worse than running time of Datta et al’s algorithm with time complexity *O*(2^(2*n*+*m*)^*τ*)

In some applications, not only a valid control strategy is needed, but also among valid control strategies (that lead to the desired state), the one with some optimality is preferred. To address this requirement we added the ability to compare valid control strategies for the proposed algorithm. Actually, the proposed algorithm finds a valid control strategy that has minimum switching of control nodes, i.e. from 1 to 0 or 0 to 1. Adding this ability to the proposed algorithm makes it more like an optimization algorithm.

The results of the comparison of our algorithm and previous algorithms implemented on a PC with Dual-Core 2.5GHz CPU, 2G RAM are depicted in Table 7.

**Table 7:** Comparison of algorithms for a control problem on the T-cell network’s receptor kinetics (Table 6) and Drosophila melanogaster’s network (Table 5).

Algorithm	T-Cell	Drosophila
Algorithm of Datta et al	Over 12 days	16.5 hours
Algorithm of transforming the graph into a rooted tree structure	Over 27 days	Over 2 days
Proposed (our) algorithm	1.83 Seconds	0.92 Seconds

## 4 Conclusion

In this paper, we proposed an algorithm for network control problem that improves the running time of previously provided algorithms. The extent of improvements and efficiency of our proposed algorithm depends on the size of the *multi-node components* of the network and also on the positioning of the control nodes or more generally, on the accessibility of both states of 0 and 1 in the same time steps for branching nodes.

Since in the proposed algorithm, the nodes are met based on a topological order, the states of the entering nodes of each node are calculated before visiting that node. As a result, in each node, it would be possible to handle states from the initial time step to the desirable time. This would cause early detection of the case that if a node is not possible to attain the desirable state, thus, there be no need to check all the nodes to time step *τ*, and the lack of a control sequence is reported early. Although the proposed algorithm shows to be efficient on real control networks, however, if all the nodes of the network are in one strongly connected components, the proposed algorithm is not performing well anymore. Handling this case could be future work.

## Supporting information

S1 Codes Implementation of the algorithms. This file contains the implementation of the provided algorithm as well as two implementations of the Datta et al algorithm. This file also contains sample inputs.
(ZIP)
