# Supplementary figures and images for: A Boolean network control algorithm guided by forward dynamic programming
Source: PLoS One. 2019 May 2;14(5):e0215449. doi: 10.1371/journal.pone.0215449 (PMC6497256; doi:10.1371/journal.pone.0215449)

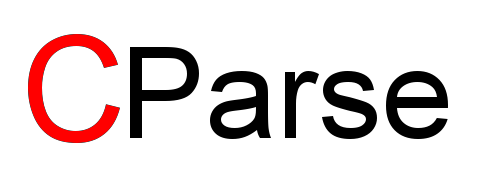

Supplement: S1 Codes — This file contains the implementation of the provided algorithm as well as two implementations of the Datta et al algorithm. This file also contains sample inputs. (ZIP) [file pone.0215449.s001.zip › S1/cparse-master/logo-cparse.png]
